# The Proteolytic Landscape of Ovarian Cancer: Applications in Nanomedicine

**DOI:** 10.3390/ijms23179981

**Published:** 2022-09-01

**Authors:** Cailin O’Connell, Sabrina VandenHeuvel, Aparna Kamat, Shreya Raghavan, Biana Godin

**Affiliations:** 1 Department of Nanomedicine, Houston Methodist Research Institute, Houston, TX 77030, USA; 2 School of Engineering Medicine, Texas A&M University, Houston, TX 77030, USA; 3 Department of Biomedical Engineering, Texas A&M University, College Station, TX 77843, USA; 4 Division of Gynecologic Oncology, Houston Methodist Hospital, Houston, TX 77030, USA; 5 Department of Obstetrics and Gynecology, Houston Methodist Hospital, Houston, TX 77030, USA; 6 Houston Methodist Neal Cancer Center, Houston, TX 77030, USA; 7 Department of Obstetrics, Gynecology, and Reproductive Sciences at McGovern Medical School-UTHealth, Houston, TX 77030, USA

**Keywords:** ovarian cancer, protease, nanomedicine, nanoparticle, matrix metalloproteinase, cathepsin, trypsin, urokinase plasminogen activator, kallikrein

## Abstract

Ovarian cancer (OvCa) is one of the leading causes of mortality globally with an overall 5-year survival of 47%. The predominant subtype of OvCa is epithelial carcinoma, which can be highly aggressive. This review launches with a summary of the clinical features of OvCa, including staging and current techniques for diagnosis and therapy. Further, the important role of proteases in OvCa progression and dissemination is described. Proteases contribute to tumor angiogenesis, remodeling of extracellular matrix, migration and invasion, major processes in OvCa pathology. Multiple proteases, such as metalloproteinases, trypsin, cathepsin and others, are overexpressed in the tumor tissue. Presence of these catabolic enzymes in OvCa tissue can be exploited for improving early diagnosis and therapeutic options in advanced cases. Nanomedicine, being on the interface of molecular and cellular scales, can be designed to be activated by proteases in the OvCa microenvironment. Various types of protease-enabled nanomedicines are described and the studies that focus on their diagnostic, therapeutic and theranostic potential are reviewed.

## 1. Ovarian Cancer: Introduction

Ovarian cancer (OvCa) is a devastating disease with a 47% 5-year-survival across stages, and is the 5th leading cause of cancer deaths in US women [1]. OvCa is a term used to describe the broad category of malignant tumors originating from the adnexa of the female reproductive system. The most common subtype of OvCa is epithelial cancer, derived from epithelial cells of the surface ovarian tissue or the fallopian tube. This category of OvCa accounts for 90% of 240,000 women worldwide diagnosed annually with OvCa [2,3].

Epithelial OvCa is divided into two main subtypes based on the clinical features and genetic evaluation: Type 1 and Type 2 tumors [4]. Type 1 OvCa represents low-grade serous, endometrioid cell, and mucinous cancers, slow-growing tumors with lower metastatic potential. Type 1 OvCa is frequently associated with mutations in BRAF, KRAS, PIK3CA, PTEN, and ARID1A. On the other hand, Type 2 OvCa, or High-Grade Serous Ovarian Cancers (HGSOC), accounts for 75% of all epithelial OvCa cases and is a highly aggressive subtype originating from serous secretory cells. HGSOC OvCa has a poor prognosis with a 5-year survival rate of less than 30% when distant metastases are involved. Table 1 summarizes the criteria of OvCa staging and 5-year-survival for all OvCa subtypes [5]. A high fraction of HGSOC (50–60%) originate from the fallopian tubes and are associated with TP53 mutations [6]. This low survival rate contrasts with early stage localized epithelial ovarian cancers with a 5-year survival of 92% [3]. Thus, early diagnosis and treatment of OvCa, particularly HGSOC, is a priority for physicians and translational cancer researchers.

The current review will focus on how researchers can target proteases in the microenvironment of OvCa to improve cancer diagnosis and therapy. More specifically, we will summarize existing tools in OvCa diagnosis, the role of various proteases in OvCa progression, and how nanomedicines may target the increased levels of proteolytic enzymes in OvCa.

## 2. Current Status in Detection and Treatment of OvCa

During the early stages, OvCa is frequently asymptomatic or has non-specific symptoms. Symptoms such as abdominal/pelvic pain, bloating, and difficulties with food consumption that persist for a prolonged period (at least 12 days in a month for up to 1 year) are 56.7% sensitive for early stage and 79.5% sensitive for advanced-stage OvCa [10]. Since the early diagnosis of OvCa can drastically improve the prognosis, several screening techniques, including cancer imaging and soluble OvCa biomarkers, have been tested in clinical and translational studies, as summarized below.

### 2.1. Clinically Used Tools and Biomarkers in OvCa Diagnosis

Transvaginal ultrasound (TVUS), one of the most frequently clinically used imaging techniques for pelvic mass evaluation, has been investigated as a potential screening tool for OvCa. Characteristics identified by TVUS include ovarian volume, cyst volume (if present), and morphology, including simple cysts ranging to more malignant septations, papillary projections, solid components, and ascites [11]. Several trials use TVUS as a screening modality for ovarian malignancy. The largest TVUS screening trial to date is the UK Collaborative Trial of OvCa Screening (UKC-TOCS) [12]. The trial included more than 200,000 patients and compared two different screening modes, TVUS and multimodal screening (MMS) (soluble biomarker Cancer/Carbohydrate antigen 125, CA125, followed by TVUS) vs. no screening. There was no significant reduction in disease-specific mortality in the MMS and TVUS groups compared to a no-screening group [13]. Thus, while a valuable tool in examining ovarian volume and architecture, TVUS alone is not considered a viable screening tool for OvCa.

A few soluble biomarkers are currently clinically available as diagnostic tools for OvCa. The most frequently used OvCa plasma biomarker, CA125, also known as mucin 16 (MUC16), is secreted from the epithelial cells and is highly expressed in patients with serous epithelial carcinoma [14]. However, increased CA125 levels are also associated with non-cancerous diseases, including heart disease and benign ovarian disorders, as well as physiologically elevated during menstruation [15,16,17]. Multiple studies show a high false-positive rate with low sensitivity and specificity of CA125 as a biomarker for OvCa [18,19]. Many reports suggest that screening using CA125 and TVUS resulted in low positive predictive values and up to a 5.8% false-positive rate [20].

Another protein of interest as a soluble biomarker for OvCa diagnosis/screening is Human Epididymis Protein 4 (HE4). HE4 is an N-glycosylated whey acidic protein with moderately improved specificity over CA125, 73% vs. 60%, in early stage OvCa [18,21,22]. Several studies suggest that the presence of HE4 is linked to the cell proliferation found in OvCa by allowing the cell cycle to progress past G0/G1; accordingly, when HE4 is added to OvCa cells in vitro, the number of cells in G2/M increases [23,24]. HE4 may also promote the PI3K/AKT pathway, responsible for cell growth, gluconeogenesis, proliferation, metastasis, and upregulating Hypoxia-Inducible Factor-1 Alpha, which promotes angiogenesis [25,26,27]. Further, HE4 was found to co-localize and interact with EGFR, an activator of the MAPK/ERK pathway, which is an important player in migration, proliferation, and avoidance of apoptosis. HE4 is likely intertwined with growth factor signaling, helping to progress OvCa disease burden [27,28]. While a promising biomarker for disease recurrence, HE4 is not yet used as a standalone screening tool or rule-out test for ovarian malignancy [29,30]. Like CA125, individual factors, such as smoking and age, individual patient health, and physiology can contribute to increased HE4 levels [31].

Several algorithms combining the data from multiple OvCa diagnostic tools have been proposed to improve assessment of disease risk. The Risk Malignancy Index (RMI), first proposed in the 1990s, uses a combination of three factors, CA125, ultrasound results, and menstrual status, to predict the risk of OvCa. In one study, the sensitivity of OvCa detection with RMI was 85%; however, while variations of the RMI formula have been proposed, sensitivity remained low unless specificity was compromised [32,33]. The Risk of Ovarian Malignancy Algorithm (ROMA) is a logarithmic formula accounting for the menstrual status of the patient and incorporates HE4 and CA125 levels, but has wide-ranging sensitivities from 76–96.7% and specificities of 74–92.5%, likely owing to laboratory technique differences in measuring HE4 and CA125 [34,35,36,37]. A modified version of the ROMA, ROMA P, which uses age rather than menstrual status, does little to improve the diagnostic value of the test, with increased specificity but lower sensitivity [37].

In conclusion, none of the existing clinically used biomarkers are sensitive or specific for early OvCa detection. Therefore, novel methods of OvCa screening and detection are highly required.

### 2.2. Current Treatment Pathways of OvCa

OvCa treatment is specific to the histologic subtype and stage at the time of diagnosis. For the purposes of this review, we will briefly describe the treatment pathway of HGSOC. After the patient presents to the clinic with new symptoms or a newly detected pelvic mass, the clinician evaluates the patient with a pelvic exam, imaging (pelvic ultrasound and/or CT/MRI of the abdomen, chest X-ray, or CT of the chest), complete blood count, liver function tests, CA125 and other tumor markers, and personal and family history. Imaging allows clinicians to assess the extent of disease and evidence of metastatic spread, which dictates the treatment plan. The decision to proceed with surgical cytoreduction versus neoadjuvant chemotherapy is determined by various factors, including the extent of disease and feasibility of surgical resection, the medical condition of the patient, and the ability to withstand surgery. Neoadjuvant chemotherapy is generally reserved for patients who are poor surgical candidates or who may benefit from pharmacologic cytoreduction prior to interval debulking surgery, such as those with extensive metastasis at the time of diagnosis [38].

Chemotherapy for OvCa typically involves a cycle of a combination of paclitaxel, a taxane that prevents microtubule formation, and carboplatin (CB), a platinum alkylating agent, every three weeks for 3-6 cycles or as tolerated [39,40]. However, combination CB and paclitaxel is associated with significant side effects, including anemia, neuropathy, and dose-limiting neutropenia [41]. Advanced or recurrent cases also benefit from the addition of bevacizumab, a recombinant antibody treatment against the vascular endothelial growth factor (VEGF) receptor, which inhibits angiogenesis [42]. Clinically, patients receiving CB plus bevacizumab treatment have an increase in progression-free-survival of 4 months versus CB alone. However, the medication is not without adverse effects, which include hypertension and gastrointestinal wall perforation [43].

Pegylated liposomal doxorubicin may also be used in combination with CB (CD) in the treatment of late-stage OvCa. One phase III noninferiority trial showed that CD was superior to CB at extending progression-free survival. However, while CD is less associated with sensory neuropathy and nonhematologic toxicity than CB, it has higher rates of mucositis, nausea, and hand-foot syndrome [44].

The recent addition of Poly(ADP-ribose) polymerase (PARP) inhibitors to the clinician’s arsenal represents one of the most significant leaps in OvCa treatment in recent years. This class of medication capitalizes on the frequent single-strand breaks of rapidly dividing cancer cells, knocking out base excision repair function, which leads to double-stranded DNA breaks (DSB). Deficient homologous recombination genes, such as BRCA1/2 mutations, prevent repair of the DSB, and the cancerous cell undergoes apoptosis [45]. Following the primary treatment modalities stated previously, maintenance therapy of Stage II-IV, recurrent, or BRCA1/2 positive disease with a PARP inhibitor prolongs progression-free survival [46,47,48].

Despite these treatment options, recurrence rates in stage III and IV patients are as high as 70–80% [49]. Response rates decline steadily with second and third-line agents, and as a result, survival for late-stage OvCa remains a losing battle. There is a great need for innovative drug delivery techniques to minimize adverse effects while maximizing anti-tumorigenic actions.

## 3. Proteases in OvCa Progression

**Figure 1:**
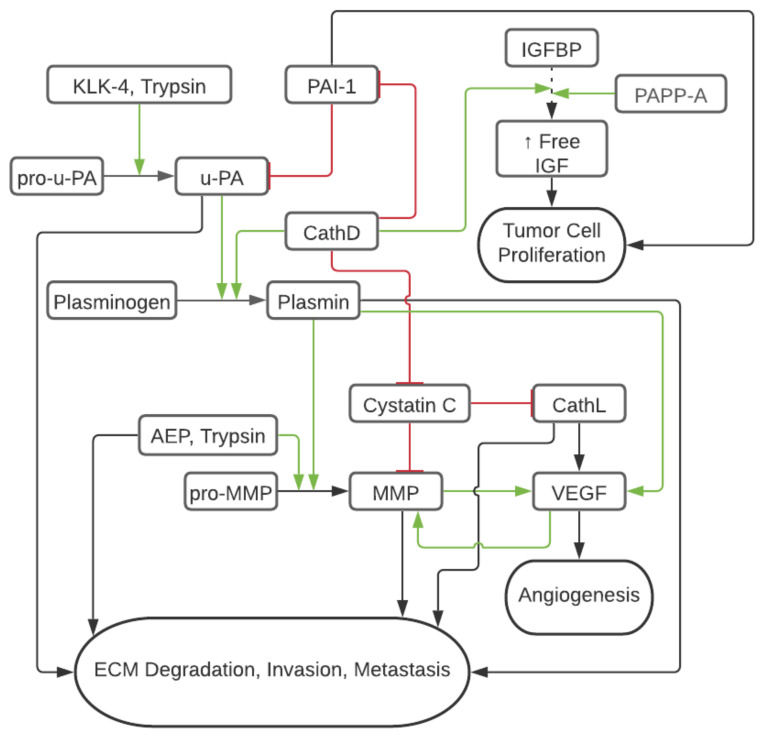
Schematic of Tumor Protease Cascade in OvCa. Red/flat line represents inhibition and decreased tumorigenic activty and green/arrow represents cleavage or activation increasing tumorigenic activity. Dashed line represents IGFBP binding of IGF and leading to decreased circulation of IGF.

Proteolytic enzymes, or proteases that physiologically support healthy tissue functions, are dysregulated in cancer, causing deleterious remodeling, tumor growth, chemoresistance, and metastasis [50]. The proteolytic environment in OvCa has been of interest for decades, with the earliest reports focusing on matrix metalloproteinases, Urokinase-Type Plasminogen Activator, and trypsin [51]. Further studies emphasized the link between proteases and clinical manifestations and tumorigenicity. Table 2 summarizes the mechanisms by which proteases can promote cancer growth and dissemination, as will be reviewed in the following section. Figure 1 summarizes the interplay between different proteases in the OvCa microenvironment.

### 3.1. Matrix Metalloproteinases

Matrix metalloproteinases (MMP) are zinc-binding protease enzymes that are highly upregulated in various cancer types [83]. MMP participate in the breakdown of the extracellular matrix (ECM), allowing tumor dissemination and metastases [84]. MMP-2, MMP-9 gelatinases, and membrane-bound MT1-MMP (aka MMP14) are overexpressed in the OvCa tumor microenvironment (TME) as compared to normal and benign ovarian tissue [85,86,87]. In one study, the expression of MMP-2 and MMP-9 in omental metastasis (Stage III and above) was significantly higher than in benign ovarian lesions and lesions of low malignant potential [87]. These findings were confirmed later by Kamat et al., who differentiated the immunohistochemical staining of MMP in the OvCa epithelium versus the OvCa stroma. MMP expression was strongly associated with OvCa aggressive characteristics, including lymph node involvement, higher stage, and presence of ascites. The shortest disease-specific survival was associated with MMP-14 strongest epithelial overexpression. These findings suggest that the presence of MMP in the stroma of the TME plays an important role in OvCa prognosis [86]. Interestingly, in another study, the increased expression of MMP-14 was associated with early stage OvCa, low CA125 levels, and inversely correlated with tumor progression, suggesting that MMP-14 may have some protective prognostic value [88]. On the other hand, a study investigating the epithelial to mesenchymal transition (EMT) in OvCa, an important hallmark of the metastatic process, correlated high co-expression of MMP-14 and CD44, but not MMP-14 alone, with poor prognosis. [89] Further publications show that aberrant activation of hedgehog signaling pathways by overexpressed Gli1 correlates with increased MMP-14, invasion, proliferation, and metastasis [90]. These findings point toward the need to shed more light on the role of MMP and especially MMP-14 in OvCa progression [91].

### 3.2. Urokinase-Type Plasminogen Activator

Urokinase-Type Plasminogen Activator (uPA) is a serine-type protease upregulated in OvCa versus healthy tissue [92]. By activating plasminogen into plasmin, uPA directly degrades ECM proteins such as fibrin, fibronectin, laminin, and proteoglycans while also activating other matrix-degrading enzymes such as pro-collagenase and MMP (Figure 1) [93,94]. The enzymatic activity of uPA is inhibited by plasminogen activator inhibitors type 1 and 2 (PAI-1 and PAI-2), which induce endocytosis and degradation of the surface uPA receptor (uPAR, thereby inhibiting the cleavage of plasminogen to plasmin at the cell surface [57]. Interestingly, PAI-1 co-expresses with IL-6 in the ascites of 83% of OvCa patients and is associated with increased chemoresistance and poor outcomes [58]. Similarly, uPA and CD44 co-expression correlate with poor OvCa prognoses [95]. Low antigen levels of uPA and PAI-1 have been found in benign ovarian tumors but significantly increase in advanced OvCa stages [87]. Both uPA and PAI-1 are overexpressed in 75% of primary OvCa cases, resulting in the downstream effects of upregulated VEGF-A and FGF2 associated with neovascularization, as well as the activation of molecular mechanisms of important OvCa progression drivers, such as AKT, mTOR, FAK, MAPK, KNK, ERK1/2, and MEK-activated phosphatidylinositol 3-kinase (PI3K) [57,96]. Downregulation of uPAR and uPA in glioblastoma cells has been shown to activate caspase 8, release cytochrome c, and cleave PARP, likely representing Fas-mediated tumor cellular apoptosis [59]. Several pharmaceutical inhibitors of the uPA/uPAR/plasmin axis and augmentation of PAI-1 have been explored. For instance, tranexamic acid, an inhibitor of the plasmin pathway, showed partial to marked reduction of ascites in 50% (6 out of 11) of primary OvCa cases and a 12-months increase in median survival in patients undergoing chemotherapy [97].

### 3.3. Trypsin

Trypsin is a serine protease best known for its role in the digestive tract as an enzyme in food digestion and absorption. In early works, it has been demonstrated in vitro that activation of pro-uPA to uPA in the TME is associated with trypsin expression (Figure 1) [60,61]. In colon carcinoma models, adding a tumor-associated trypsin inhibitor restricted ECM degradation by 57%, suggesting that trypsin plays a role in the metastatic advancement of cancer [98]. Later work reinforced the association of trypsin overexpression with OvCa as compared to ovarian tumors of low malignant potential and normal ovarian tissue, which showed no antibody staining for the protease [62]. Trypsin activates pro-MMP-9, but not pro-MMP-2, in ovarian tumor cyst fluids in vivo, suggesting that trypsin has a role in regulating MMP activity in the OvCa microenvironment (Figure 1) [63]. In addition, trypsin activates protease-activated receptor 2 (PAR2), a transmembrane receptor that promotes cancer cell proliferation by ERK phosphorylation (Figure 1) [99].

### 3.4. Pregnancy-Associated Plasma Protein A

Pregnancy-associated plasma protein-A (PAPP-A) is a zinc metalloproteinase, first identified in 1974, and responsible for Insulin-like Growth Factor (IGF)-dependent proteolysis of IGF binding protein 4 (IGFBP4) (Figure 1) [65,66]. The cleavage of the IGFBP4 results in the increased circulation of IGF-I and IGF-II, allowing for increased ability of (1) IGF-I to bind IGF-IR, furthering proliferation, invasion, metastasis, and (2) IGF-II to bind Insulin Receptor Isoform-A stimulating additional cell proliferation pathways [67,68,69]. Additionally, PAPP-A effects are augmented by associating with the cell surface proteoglycans of both the secreting and neighboring cells, allowing for autocrine and paracrine IGF/IGF-IR signaling [100,101]. In OvCa models, downregulation of PAPP-A mRNA showed a decreased cell burden in vitro, while upregulation of PAPP-A expression resulted in an increased incidence of OvCa metastasis in vivo [102]. Further, Boldt & Conover showed that in vivo wild-type PAPP-A induced OvCa vascularization and accelerated tumor growth in SKOV3 murine xenografts. Moderate PAPP-A expression induced earlier tumorigenesis and solid tumor growth, while high PAPP-A expression correlated with increased OvCa invasiveness [103]. In clinical studies, OvCa tumor tissue and ascites express PAPP-A, IGFBP-4, IGF-I, and II, with ascites levels greater than 50 times that of the serum levels, which were found to be equal in both cancer and healthy patients [104]. Thus, while PAPP-A may not represent a serum biomarker of interest for OvCa detection, the high levels of PAPP-A in the TME play a pivotal role in tumorigenesis. Thus, PAPP-A is an attractive therapeutic target. For instance, therapy with a monoclonal antibody targeting PAPP-A in immunocompromised mice with patient-derived OvCa xenografts reduced tumor growth, ascites accumulation, and reversed platinum chemoresistance [105].

### 3.5. Cathepsin L

Overexpression of cathepsin L (CathL), a lysosomal cysteine protease affecting cell proliferation, angiogenesis, inflammation, and ECM remodeling, has also been reported in OvCa [70]. Sui et al. found that of 58 OvCa patient samples, 41 expressed three times the normal adjacent tissue level of CathL. In vitro studies with SKOV3 paclitaxel sensitive and resistant OvCa cells showed that after paclitaxel treatment, levels of CathL decreased in paclitaxel sensitive cells but remained elevated in the paclitaxel-resistant cells. After CathL knockdown in the SKOV3 paclitaxel-resistant cells, cellular apoptosis increased by a factor of 3, cellular migration was reduced by a factor of 1/3, and cellular invasion was reduced by a factor of 1/2 [71]. Additionally, CathL contributes to angiogenesis by increasing expression of galectin 1 (Gal1) mRNA, leading to downstream activation of the MEK/ERK1/2 pathways, verified with immunohistochemistry of omental metastasis demonstrating a positive correlation between Gal1 and vascular proliferation [106].These results have been validated in murine models in which knockdown of CathL resulted in reduced tumor development [107].

### 3.6. Cathepsin D

Cathepsin D (CathD) is a lysosomal aspartic endoproteinase that is pathologically upregulated in many malignancies, including OvCa, and associated with metastatic disease [70,108,109]. The exact mechanism by which CathD is secreted extracellularly from the lysosomes into the body fluids has not yet been fully elucidated. One proposed mechanism suggests estradiol-driven downregulation of the Mannose-6-Phosphate receptor gene leading to a lack of binding sites available in the lysosome and subsequent secretion of excessive enzyme [99,110]. CathD is also upregulated in a number of other inflammatory conditions, including atherosclerosis, obesity, and other malignancies. In breast cancer, CathD is associated with poor outcomes [111,112]. Interestingly, CathD contributes to the cleavage of plasminogen and IGFBP and, therefore, the activity of uPA and PAPP-A, respectively (Figure 1) [72]. The roles of CathD in oncogenesis are vast and both proteolytic and non-proteolytic. For instance, CathD has been shown to non-proteolytically activate ERK1/2 to facilitate HGSOC proliferation of omental metastasis, as well as ERK1/2 and AKT to facilitate migration of the cancerous omental cells [113]. On the other hand, the proteolytic activity of CathD is known to be pH dependent. However, while the ideal pH of CathD to exhibit proteolytic activity is around 3.5, it has been found in breast cancer models to cleave cystatin C, a metalloproteinase and cysteine cathepsin inhibitor, at pH 5.5-6.8, the pH of the TME [74]. Cystatin C is an inhibitor of both metalloproteinases and cysteine cathepsin activity, such as CathL, which suggests the cathepsins play an essential role in the protease network to allow the pro-metastatic effects of MMP to take effect (Figure 1). Additional work in breast cells has demonstrated that CathD has the dual action of increasing the activity of uPA and decreasing the activity of its inhibitor PAI-1 in the acidic pH 6.6 found in the hypoxic TME [73]. Besides these changes in the enzyme functionality in the hypoxic tumor environment, CathD and its precursor aid in ECM degradation, promoting the angioproliferative basic fibroblast growth factor pathway, which further potentiates the proliferative and metastatic effects of the enzyme. Interestingly, in work by Vangala et al., CathD enzymatic activity was the basis of mesenchymal stem cells (MSC) chemoattraction in both colon and breast cell models, and MSC movement is reduced by inhibiting CathD activity [75]. Notably, while the presence of MSC cells in the TME has mixed pro- and anti-tumorigenic activity due to a large amount of cytokine released, MSCs inhibit the cytotoxic activity of natural killer cells [76,114]. Thus, CathD and pro-CathD have multiple enzymatic effects in the TME and potentiate the effects of uPA, PAPP-A, CathL, and MMP to promote metastatic and angioproliferative activity.

### 3.7. Kallikrein-Related Peptidases

Kallikrein-related peptidases (KLK) are a part of the serine protease class of the human degradome and are physiologically present in healthy tissues. The ovaries naturally express KLK-10 and KLK-11 at levels of 4.4 and 0.5 reads per kilobase, respectively. However, data from the cancer genome atlas of 373 OvCa patients show a strong expression of KLK-5–8 and KLK-10–11 in OvCa [115]. While KLK-5–7 and KLK-10 are associated with poor progression-free and overall survival, the mechanism associated with the prognostic role of KLK-8 is not yet well understood [115].

KLK-11 is thought to be a protective factor and is associated with prolonged progression-free survival and overall survival [116]. KLK-4–7 appear to play a role in the function of both primary tumor growth and peritoneal metastasis, chemo-resistance, survival, and proliferation. KLK-4 has been shown to cleave and activate uPA, KLK-5, and KLK-6, setting off a cascade of proteases that allows for invasion, metastasis, and chemoresistance [77]. Therefore, KLK-4 likely participates in the protease web by activating pro-uPA to uPA to allow it to assert its downstream effects (Figure 1). Additionally, KLK-4–7 have been shown in vivo by a xenograft model to increase the expression of Transforming Growth Factor β-1 and L1 Cell Adhesion Molecule, contributing to EMT and metastasis [78].

### 3.8. Asparagine Endopeptidase (Legumain)

Asparagine Endopeptidase (AEP) is a part of the cysteine protease class of the human degradome. It is predominantly located within the late endosomes and lysosomes within human immune cells. AEP plays an important role in the self-tolerance processing of self and foreign proteins for presenting MHC II on T cells. Loss of the self-tolerance function of AEP has pathologic implications in multiple sclerosis, in which exaggerated AEP protease activity leads to cleavage of myelin basic protein peptides causing failed immune tolerance in the thymus [117]. AEP is known to be expressed in human peritoneal mesothelial cells (HPMC) in addition to OvCa, and has been found to co-localize with integrin α5β1, which enables the recognition of fibronectin as well as binding with AEP [79,80]. Once an integrin α5β1/AEP complex forms, it is secreted by the OvCa into the ascites as exosomes and readily taken up by HMPC. This action promotes peritoneal metastasis via proliferation of HMPC via the FAK/AKT/ERK signaling pathway and additionally encourages EMT [81]. This finding and proposed pathway are reinforced by clinical correlation studies which associate increased AEP expression with higher stage and ascites positive for tumor cells. Further, the iTRAQ proteomics approach revealed that AEP is upregulated by a factor of five in OvCa, and activates MMP-2 and MMP-9, augmenting their role in ECM destruction (Figure 1) [82]. In sum, it appears the primary role by which AEP contributes to cancer progression in OvCa is facilitating the EMT to allow for the spread of abdominal metastasis.

## 4. Protease Targeting Nanomedicine in OvCa

Due to the limitations of current clinically used OvCa biomarkers and therapeutics, other approaches are actively being pursued. In recent years, particular interest has been given to the tumor protease microenvironment as a tool to be leveraged in cancer nanotherapeutics, nanodiagnostics and nanotheranostics [83,84]. Nanoparticles (NP) can be designed to be activated upon exposure to higher concentrations of a specific protease. Characterization of protease activity and their substrates in the TME, as summarized above, can provide a roadmap to enzyme targets and consequent nanomedicine design [118]. Of particular interest in protease-based nanomedicines is the inhibition of pro-tumorigenic protease activity and nano pro-drug approaches, which use NP as a repository to reduce chemotherapeutic toxicity in healthy tissue and concentrate apoptotic activity in tumor cells [119].

Additionally, nanomedicine provides a useful platform for combined diagnostic and therapeutic approaches because of the potential to encapsulate and conjugate multiple substrates to the NP carrier system [120]. There is a great potential to utilize protease-targeted nanomedicine for early diagnosis and therapy of OvCa [121,122,123,124]. Below, we review NP structures and design characteristics while discussing models and applications of the overexpressed proteolytic enzymes for OvCa nanomedicines.

### 4.1. Overview of Nanoparticle Designs

The National Institutes of Health defines nanomedicine as medical interventions that can participate in the curative and reparative treatment of human tissues in a “highly specific” manner [125]. This description captures the advantages that materials at or below the micron scale can provide in the medical field. Because NP are at the juncture of micron (cells) and angstrom (proteins, DNA, and other macromolecular building blocks) scales, they can precisely intervene in numerous biological processes. Importantly, NP accumulate in solid organ tumors owing to the increased vascularization and lymphatic leakage in the microenvironment, a phenomenon referred to as enhanced permeability and retention (EPR), first described by Maeda in 1986 [126]. The basic principle of the EPR effect is that new or injured endothelium have gaps sufficient for NP to permeate [127,128]. In addition to EPR changes in carcinogenesis, impaired blood flow, and endothelial structure changes are among the pathological features in multiple neurodegenerative and neuroinflammatory diseases, as well as in inflammatory and infectious diseases [129,130]. While EPR is affected by tumor heterogeneity, this phenomenon nonetheless makes NP an attractive vehicle for delivery of chemotherapy, RNAi, or immunotherapy to achieve greater concentration while minimizing off-target effects [131].

**Figure 2:**
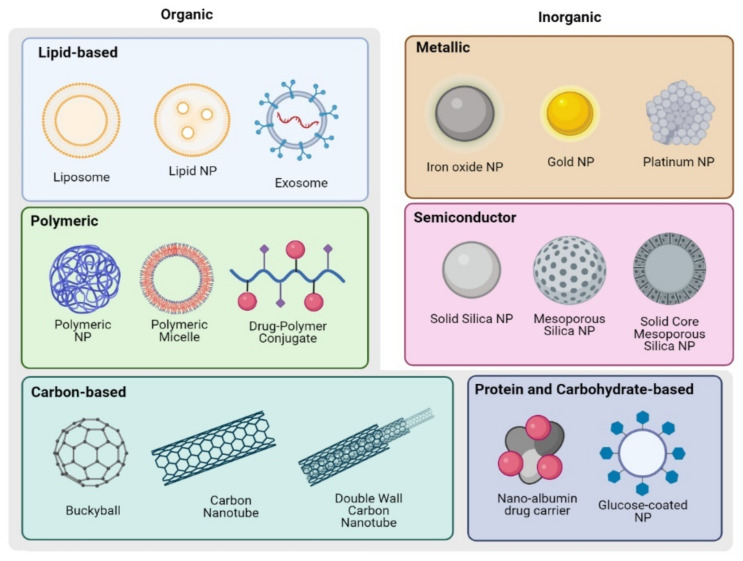
Schematics of NP structures used in OvCa detection and treatment. Created with BioRender.com.

To better understand protease activity targeted NP interventions, we will briefly review the various types of NP used in OvCa (Figure 2). Lipid, protein, polymeric, carbon, and sugar-based NP are examples of organic NP, whereas inorganic NP can be semi-metal or metal-based. Physical characteristics inherent to the type of NP such as the size of the particle, surface charge and hydrophobicity, stiffness, and geometry influence the pharmacokinetics, tissue biodistribution and safety profiles and may be altered to allow for efficient delivery of payload encapsulated in the NP [132,133,134]. The basic NP structures can further be actively to specific cells and structures in the TME by conjugating targeting moieties to the surface of the NP, such as antibodies [135], peptides [136,137], and nucleic-acid-based moieties [138,139].

#### 4.1.1. Organic NP

Organic NP include lipid-based NP, polymer-based NP, protein or carbohydrate NP, and carbon NP. Each material offers unique benefits, and there are numerous examples of applications in oncology and beyond, as discussed below.

##### Lipid-Based NP

Lipid NP represent the most widely utilized nanostructure currently utilized in clinical treatment of cancer [140]. Close to three decades ago, doxorubicin (DOX) and amphotericin B liposomes were the first nanomedicines introduced to clinical practice, demonstrating their value in treating disease as well as preventing unwanted side effects associated with the free drugs administration [141,142]. Lipid NP have also recently been used as a delivery method for mRNA vaccines against the COVID-19 virus during the pandemic [143]. While lipid NP and liposomes are similar in their composition (phospholipids and cholesterol in aqueous environment), their structure differs. Liposomes are characterized by the presence of aqueous core surrounded by lipid biolayers, while, lipid NP self-assemble without aqueous core, owing to the electrostatic interaction between positively charged phospholipids and negatively charged payload (nucleic acids). The majority of lipid-based nanomedicines used clinically exploit the tumor bed’s increased vascularity and EPR phenomena for tumor accumulation [131,144].

Other lipid NP are solid lipid NP and nanostructured lipid carriers, which incorporate lipids that are solid at room temperature. Both of these structures are utilized as vehicles for primarily lipid-soluble drugs in addition to finding use in the cosmetics industry [145,146]. Additionally, ethosomes and transfersomes, are used for transdermal applications by leveraging alcohols and cholates in the lipid bilayer, respectively, to enable a malleable structure that can permeate the skin barrier [147,148,149,150]. In the presence of a phase stabilizer, cubosomes were made using amphiphilic lipids such as glyceryl monooleate and phytantriol to increase the surface area of particles while lowering viscosity, in order to better allow for drug delivery to the mucosa, enterally, transdermally, and parenterally [151].

Biomimetic lipid-based NP such as nanoghosts and leukosomes incorporate components of native cell membranes into the NP structure and have been shown to modulate the immune system [152,153]. Physiologic NP called exosomes can be produced by the cells and the production can be scaled to target the disease based on natural trophism, including OvCa [154,155]. Similarly, physiologic extracellular vesicles have been discussed as possible carrier systems for therapeutic delivery [156].

##### Other Organic NP

Polymeric NP, polymeric micelles, lipid-core NP, and drug-polymer conjugates are all examples of polymer-based NP [157,158]. In one early study, entrapment of fluorescein in polyacrylamide NP allowed for the accumulation of the dye in lysosomes, an atypical delivery site, which demonstrated the ability of polymer NP to serve as a means of guiding payload to a delivery site [159]. Drug-polymer conjugates have been used in the clinic for several decades, and leverage linker molecules that cleave releasing therapeutics in the TME [160]. The benefit of using polymeric nanocarriers is the ability to fine-tune the properties based on the building blocks of the polymers to carry both hydrophilic and lipophilic drugs [161]. In particular, the use of particle replication in nonwetting templates (PRINT) has been utilized to create size and shape specific poly lactic-co-glycolic acid NP capable of delivering a high chemotherapeutic payload to tumors, which spare healthy tissue with tumor receptor targeting and intracellular activation due to pH drop in endosomes or reduction by the cytosol [162]. Medications such as Apeala, a polymeric form of paclitaxel for ovarian cancer, represent the ability to translate polymer formulations from the benchtop to the infusion center, with more polymer based medications currently under clinical trial [140]. Carbohydrate NP are characterized by their high solubility in aqueous environments and protein repellency. The latter prevents opsonization and clearing by macrophages. Various sugar monomers have been utilized for carbohydrate NP, including glucose, mannose, galactose, and trehalose [163]. Protein and sugar-based NP are biodegradable and are considered to have low toxicity. The most famous clinical example of a protein-based nanostructure is Abraxane, a nano-albumin bound formulation of paclitaxel approved for use in 2005 [164]. Aside from albumin, silk fibroin, gelatin, and lipoprotein may also be utilized as substrates for protein NP creation [165,166,167].

##### Carbon Nanostructures

Carbon has a variety of atomic structures, with the earliest known naturally occurring allotropes being diamonds and graphite. In 1985, Kroto et al. revolutionized the field of carbon nanomedicine by showing that laser irradiation of graphite produced an allotrope of carbon using 60 atoms (C60) called icosahedral buckminsterfullerene, composed of 20 hexagons and 12 pentagons, colloquially known as “buckyballs” [168]. Fullerenes are the third allotrope of carbon which take the aforementioned spherical shape, making them attractive vehicles for nanotherapeutics [169].

Carbon nanotubes (CNT), hexagonal helices forming a symmetric cylinder of carbon atoms, were discovered in 1991 by Ijima [170]. One shortcoming of both carbon NP is they lack the ability to disperse in water. However, this hydrophobicity can be overcome with suspension in co-solvents, the addition of a water-soluble group, or encapsulation [164,171,172]. In the literature, there are numerous examples of carbon structures being utilized to detect and treat OvCa. Multiwalled carbon nanotubes (MWCNT) with dielectric sensing surfaces have been shown useful in the detection of squamous cell carcinoma antigen, which is highly expressed in OvCa even at early stages [173]. The presence of CNT alone induces apoptosis of the cancer cells and makes them more susceptible to subsequent paclitaxel treatment [174]. MWCNT can similarly prevent metastasis by promoting cell apoptosis and interrupting the actin cytoskeleton as well as impairing mitochondria function by stalling the electron transport chain [175].

Graphene oxide (GO) nanosheets are a newer class of nanostructure that employ sp^2^ hybridized carbon atoms to create hydrophilic sheets of hexagonal lattice utilized in biosensing applications [176].The high surface area of this nanostructure and ionic interaction potential makes it a particularly attractive method for adsorbing deleterious enzymes in the TME, and has been accordingly utilized to bind CathD and CathL [177]. Such work has clear implications in attenuating the metastatic activity of OvCa by removing enzymatic drivers of metastasis from the TME.

#### 4.1.2. Inorganic NP

Inorganic, metal or semi-conductor-based, NP offer unique magnetic and optical properties that can be utilized for cancer imaging. Additionally, NP from this category have been investigated as OvCa therapies based on their ability to carry molecular therapeutics, active targeting by means of external forces (e.g., magnetic), hyperthermia induction and production of anti-cancer reactive oxygen species (ROS) generation.

##### Metallic NP

Metallic NP offer unique features which include the ability to respond to magnetic stimuli, act as conductors for heat, and serve as contrast elements in imaging. Superparamagnetic iron oxide NP (SPION) have been used in the clinic for Magnetic Resonance Imaging (MRI) of the liver malignancies for the past two decades and for magnetic hyperthermia of brain tumors [178,179]. SPION are comprised of a diverse range of cores, sizes, and coatings which allow for biocompatibility and conjugation of ligands [180]. Because the iron oxide core is magnetic, it can be used as a contrast agent in MRI and to target chemotherapeutics and gene therapies under the guidance of a magnetic field [181]. Further, SPION cause ferroptosis of OvCa cells and induce oxidative stress, which can reduce chemoresistance and lead to tumor death [182,183]. Despite solid data to support the efficacy of SPION for OvCa therapy and early diagnosis, more translational work is needed [184].

Gold (Au) is a noble metal that can be assembled into NP (AuNP). AuNP can be produced through bottom-up or top-down approaches, yielding various shapes and sizes [185]. Surface modifications of AuNP include conjugation to fluorescent probes, therapeutics, protein-based ligands (e.g., antibodies, enzymes, etc.) nucleic acid-based ligands to accomplish a biological task. AuNP can have practical applications in OvCa detection enhancing the capability to quantify CA125 levels in human plasma samples. Cysteamine capping of AuNP allows for fixation of CA125 antibodies and layering over graphite electrochemical immunosensors. This technique is more sensitive and efficient than existing immunohistochemical methods of detecting CA125 [186]. In addition, similar to SPION, AuNP can create free radicals that lead to cancer-killing properties [187].

Further, AuNP have been shown to prevent resistance to CP by downregulating the Akt and NF-kB signaling axis in OvCa, inhibiting EMT, and slowing tumor growth [188]. AuNP can also modulate inflammatory pathways in OvCa and TME cells to slow activation of cancer-associated fibroblasts linked to treatment resistance and disease progression [173]. These properties likely contribute to the decreased expression of Ki-67, a proliferation marker, in DOX conjugated to AuNP versus free DOX [189].

Platinum (Pt) is a catalytic metal widely used in the automotive and jewelry industry. Additionally, Pt has a long held a place in oncology, as Pt-agent CP was fortuitously identified as a product of Pt electrolysis that inhibited bacteria growth in 1965, and today along with its analogue CB is a commonly used chemotherapeutic for OvCa [190,191]. Owing to its radical scavenging properties, platinum NP (PtNP) have wide-ranging applications in oncology, including diagnostics as imaging or redox probes, adjuvants to chemotherapy, and phototherapy treatments [192]. Leveraging the peroxidase-like activity of of Pt, PtNP applied to GO and functionalized to breast cancer with folic acid has been shown to allow for visually detectable colorimetric changes when exposed to breast cancer cells versus healthy tissues to allow for detection of cancer [193]. Plant extract-derived PtNP were shown in one study to slow the migration of MCF-7 cells, a breast cancer cell line, in addition to inhibiting cellular proliferation [194]. PtNP induce apoptosis in ovarian teratocarcinoma in vitro models while sparing normal human normal peripheral blood mononucleocytes, as opposed to CP which also reduces normal cell viability [195]. This finding demonstrates the possibility that PtNP may have a more favorable off-target side effect profile when compared to traditional chemotherapeutics.

##### Semiconductor: Silica and Silicon

Silicon-based materials and their oxides (e.g., silica) possess unique physical and chemical properties that can explain their vast use in various industries, including medicine. Non-porous or mesoporous silicon and silica NP (SiNP) can be functionalized to disperse in aqueous solutions and exhibit biocompatibility, making them an attractive tool for biomedical uses [196]. In addition, the large surface area and modifiable pore size allow for fine-tuning the drug release characteristics as well as targeting ligands conjugation for more specific drug delivery [197].

SiNP of varying structures can be employed for OvCa detection and treatment. The wormhole mesoporous silica structure has been leveraged for its prolonged half-life and modifiable drug release profiles. Pairing the SiNP with pH-sensitive insertion peptides allowed for the delivery of infrared dye and targeting of carboplatin-loaded SiNP to the acidic OvCa environment [198]. In another work, circulating tumor cells have been detected at concentrations of 100 OvCa cells in just 50 μL of whole blood by conjugating Mucin-1 antibodies to the surface of fluorescent magnetic SiNP, representing the potential use of SiNP in early ovarian cancer detection using patient blood samples [199]. Recently, dendritic SiNP have been utilized to potentiate the diagnostic value of CA125, cancer embryonic antigen (CEA), and alpha-fetoprotein (AFP) by providing a multiplexed barcode sensor that allows for sensitive and high throughput assays of tumor markers versus standard immunochemistry [200].

SiNP have been investigated for OvCa therapy as drug and gene delivery carriers as well as radioisotope carriers for targeted radiation therapy [201,202]. While SiNP alone can be cytotoxic to cancer cells, addition of donor groups or chemotherapeutics increases the efficacy of the particles and intervention [203,204,205]. Several reports also focus on the potential of porous silicon nanovectors in OvCa therapy [206]. However, Silicon based NP have not reached the clinic yet. Their safety profile depends on multiple factors including size, shape and surface modifications [207].

### 4.2. Disease Models of the Protease Environment

Accurate modeling of the protease environment is highly dependent on the ability of a model to mimic complex interactions between cancer cells, immune cells, and the surrounding ECM. Below, we summarize various approaches to replicate OvCa TME in vitro and in vivo to better understand and leverage the characteristic proteases.

#### 4.2.1. 2D In Vitro Models

For years, cancer research and therapeutic discovery have relied heavily on two-dimensional (2D) cell culture models. Despite limitations in representing certain aspects of the TME, these tools are indispensable. 2D models can be highly beneficial considering their simplicity and reproducibility, providing important insight into cancer cell behavior and analysis of proteolytic activity. They are especially useful in studies that evaluate mechanisms through molecular and therapeutic pathways, including agents like siRNAto knockdown genes, anti-cancer drugs, or other TME-modulating agents [208,209,210,211]. These models can consistently control such factors, which indicate their value in mechanistic discovery and preliminary drug screening.

As biomarkers for tumor progression and metastasis, MMP have been extensively studied in 2D models to understand their role in assisting cell migration through proteolytic matrix degradation. Wang et al. performed MMP siRNA-mediated gene knockdown on OvCa cell lines, showing a correlated decrease in MMP protein expression via Western Blotting, cell migration via Transwell plate assays, and drug resistance evaluated with flow cytometry [208].

Additionally, proteome inhibitors have been used in 2D models to study drug resistance and cancer metastasis-indicating gene expression. Zhang et al. implemented calpeptin treatment to inhibit calpain proteolytic activity in OvCa cells. This study applied calpain inhibition and drug treatment to various degrees correlating with clinical tumor samples. The effects of chemotherapy drugs CP and CB on protein expression and cell proliferation were evaluated, and the results indicated the involvement of proteolytic activity in chemoresistance and EMT, which correlate to cancer metastasis [209].

Despite the value of 2D models in fundamental cancer discovery presented here, their limitations remain. Monolayer culture cannot replicate the complicated 3D architecture and interactions present in the TME, creating a need for biomimetic models to understand more complex processes involved in disease progression.

#### 4.2.2. 3D In Vitro Models

The addition of the third dimension in cancer modeling more accurately represents the TME than traditional monolayer culture. In these models, crucial aspects of the TME can be mimicked, such as the aggregation and morphology of the cells as well as their cell–cell and cell-matrix interactions, which can strongly influence cell behavior and disease progression.

Some popular 3D cancer models include scaffold-free spheroids [212,213], hydrogels and other biomaterials-based models [213,214,215,216,217,218,219], and microfluidic devices [219]. Scaffold-free spheroids provide a unique opportunity to isolate and study cellular interactions, including the importance of cancer-associated fibroblasts (CAF) in protease activity and cancer metastasis. CAF are the most abundant cell type in the TME and play a variety of significant roles in cancer progression, including signaling through cell–cell adhesions and cytokine production to create a pro-tumoral environment and ECM degradation and remodeling through proteolytic activity which promotes metastasis [220]. Specifically, CAF are associated with an upregulation of serine proteases and MMP and cancer cell stromal invasion [213]. Park et al. co-cultured CAF with adenocarcinoma in low-attachment round bottom culture plates forming scaffold-free spheroids to evaluate the production of protease FAP-α and the direct impact of CAF on spheroid formation [213]. Cancer cells can also be embedded in hydrogels or other materials to allow 3D tumor spheroid formation and mimic native TME ECM interactions. These materials are advantageous in studying proteases to understand the architectural nuances of ECM degradation by cell-secreted proteases. Some examples of these biomaterials include Matrigel [213,214,219], natural and synthetic hydrogels (collagen [214,219], gelatin [216], polyethylene glycol (PEG) [215,216], silk [218], etc.) and methylcellulose [216]. From there, matrix stiffness and other environmental factors such as protease activity/inhibition can be modulated to understand their effects on spheroid growth, colonization, and resistance to chemotherapy [215]. Finally, microfluidic devices are complex models which often incorporate ECM components, hydrogels, or other matrices and cell lines. They can be tuned to mimic the TME and have been designed and implemented to study migration patterns and proteolytic ECM remodeling [219].

#### 4.2.3. In Vivo Models

Due to the complex nature of OvCa and involvement of multiple tissues and organs, in vitro models are frequently unable to fully recapitulate the pathological processes involved in OvCa progression and dissemination. Therefore, various in vivo OvCa models are essential for translating protease-powered nanomedicines from the benchtop to the bedside. Proper model selection is vital in pursuing and evaluating new therapeutics, diagnostics and theranostics.

Murine models of OvCa are the most frequently investigated in vivo models. These include xenografts, in which human OvCa cells or patient-derived xenografts (PDX) are implanted into immunocompromised mice, syngeneic models, in which murine OvCa cells are injected into immunocompetent mice, and Genetically Engineered Mouse Models (GEMM) [221]. Both xenograft and syngeneic models can be established using intraperitoneal (IP) or subcutaneous (SC) injections. Peritoneal injection of OvCa cells mimics disseminated disease. Orthotopic OvCa models are established through a surgical procedure, in which tumor cells are injected into the ovarian bursa, therefore modeling early stages of tumorigenesis [222].OvCa PDX are taken from patient material, including primary tumor tissue or ascites in patients with known ovarian disease, and can effectively model tumor heterogeneity and chemoresistance [223]. The stroma and vasculature present in the original patient sample may not be maintained in the PDX model, making modeling protease-mediated processes hard to follow [224].To compensate for the immunosuppressed nature of the model, the addition of human tumor-associated cells, including fibroblasts and immune cells, can increase the fidelity of the protease-based studies [225,226]. PDX models are advantageous, due to their heterogenic nature resembling the clinically observed disease [227,228]. Interestingly, OvCa cells in PDX exhibit only mild genetic drift upon implantation into mouse models [228]. However, repeated passages of cell lines are well known to cause genetic alterations that result in inaccurate models of the disease that the cells originate from in advanced passages [229]. Therefore, only early passages should be used in established cell lines and genetic profile/protease expression should be verified.

Syngeneic mouse models involve injecting murine-derived tumor cells into immunocompetent mice. The most used syngeneic model is ID8 OvCa cells implanted in the C57BL/6 mice [230]. In recent years, syngeneic models have been further improved by CRISPR/Cas9 gene editing to inactive TP53 and BRCA, creating a tumor that is genetically similar to OvCa in humans [231]. Syngeneic models have an intact immune system, which is an advantage over xenograft models [232]. Further, a syngeneic ID8 model with secreted protein, acidic, and rich in cysteine (SPARC) matricellular protein knockout attenuates the VEGF-integrin-MMP signaling axis, suggesting that ID8 grafts are a valid model for protease activity [232].

GEMM target cancer drivers to promote in vivo spontaneous tumor growth in immunocompetent animals [233]. GEMM allowed for the discovery of critical genetic drivers in oncogenesis while also approaching histologic similarity to HGSOC [234,235]. While interspecies immune system variability exists, syngeneic mouse models with tumors derived from GEMM have been shown to replicate the immune environment of human tumors, making this hybrid model an attractive testing ground for protease nanomedicines [236].

Another in vivo non-murine model is the chicken egg chorioallantoic membrane (CAM), a highly vascularized extra-embryonic structure. CAM and nutrient-rich environments allow for rapid tumor formation. Additionally, this low-cost model has excellent ECM development, making it an attractive model for investigating protease activity contributing to EMT and metastasis in the OvCa environment [237]. A CAM model of OvCa was utilized in testing the application of mesoporous SiNP to administer DOX and showed the elimination of tumors in the model in 3 days, demonstrating the value of this model in testing therapeutics [238].

### 4.3. Cancer Detection Utilizing Protease Activated Nanomaterials

Synthetic biomarkers (SB) are diagnostic nanosensors that can leverage and amplify pathological signals, such as the upregulation of protease in the TME. SB can be applied in multiplexed setups based on proteases known to be up or downregulated in a specific cancer type [239]. Protease-cleavable fluorescently tagged peptides can be encapsulated within or conjugated to NP surface and released by proteases at the target site.

Since OvCa has poor outcomes in later stages, nanomedicine detection strategies leveraging the protease environment are of great interest. Table 3 summarizes studies exploring SB in OvCa early detection and protease-activated therapeutics, discussed in the subsequent section. In an orthotopic murine model utilizing the OVCAR8 cell line, MMP-9 sensitive SPION with tumor penetrating ligand outperformed the clinical biomarker HE4 at the early time point of 2 weeks. This platform detected tumors less than 5 mm in diameter at a volume of 2.4 times less than that of HE4, representing the potential for 5 months earlier diagnosis versus HE4 [121]. Further, cancer cell protease expression can be induced using synthetic biology techniques to create more specific protease targets, by leveraging OvCa associated molecular changes to amplify reporter readout. In one study, Tobacco Etch Virus (TEV) protease expression was transduced to OVCAR8 cells via transcription factors (a) endoribonuclease Cys4 gene and (b) miRNA-based self-inhibitory gene. These factors acted on the OvCa specific synthetic promoters (a) S(E2F1)P and (b) S(cMyc)P, which triggered the expression of TEV protease only when both promoters were present. Subsequently, a 40 kDa eight-arm PEG conjugated to TEV substrate (Biotin-eGvndneeGffsar-K(FAM)-dGGENLYFQGGGC) with reporter molecule (glutamate fibrinopeptide B) or fluorescent marker was systemically administered to tumor-bearing mice and readout recorded by urine immunoassay or blood fluorescence, respectively. Although an interesting concept, reporters for TEV protease, indicating the OvCa, were detected at only a 1.6 fold change (*p* = 0.0078) and 1.2 fold change (*p* = 0.0027) in the urine and blood samples of OvCa mice versus healthy mice, respectively, representing a change that may be challenging to detect in clinical settings [240]. Nonetheless, synthetic biology may have a unique role in amplifying protease diagnostics for detection of OvCa.

In addition to purely diagnostic NP, the protease-activity-driven NP offer a unique opportunity to combine diagnostic and therapeutic features, enabling a theranostic approach for tumor management. For instance, liposomal core, poly-l-arginine (PLR), luciferase siRNA, and propargyl-modified poly-l-aspartate (pPLD) layer-by-layer NP conjugated to MMP-9 substrate were able to detect OvCa xenografts at an average 36 mm^3^ volume and reduce luciferase activity by almost half [122]. This work demonstrates the opportunity to not only detect disease earlier but intervene at the time of detection to potentially slow the disease course.

Protease activity has also been measured by cleaved reporter peptides to reflect the proteolytic tumor profiles in colorectal cancer, lung adenocarcinoma, and prostate cancer [121,241,242]. Urine color changing AuNP clusters that are renally cleared only after MMP-9 cleavage reduces the size of system have shown promising results in colorectal cancer mouse models, giving a binary response that is appealing for use as a screening test that could be employed clinically [243]. In another study, SPION conjugated to a protease cleavable probe were able to detect protease activity in early stage breast cancer and pancreatic cancer at sub-femtomolar concentrations in patient sera [244,245]. AuNP activated by MMP-2 have also been utilized to characterize protease activity and exhibit retention at the cleavage site in a glioblastoma mouse model, which like OvCa upregulates MMP-2 [246]. The work done to successfully detect other cancer types by leveraging the protease environment can likely be translated to early screening and diagnostics of OvCa.

### 4.4. Leveraging Protease Activity for Targeted Therapy

The ability to target and interfere with protease activity to optimize chemotherapeutic delivery and minimize off-target effects is being intensively investigated [83,245,247]. Below we discuss protease-activity-driven nanomedicine interventions in OvCa and other cancer types.

#### 4.4.1. Protease-Activity Driven Nanotherapy in OvCa

The use of protease-sensitive NP to preferentially deliver chemotherapeutics to the OvCa microenvironment increases cancer-killing potential, while decreasing the off-target effects (Table 3). In a recent paper, DOX prodrug conjugated to Cathepsin B-specific cleavable peptide was utilized to create self-assembling NP (PNP) for intraperitoneal chemotherapy of advanced-stage OvCa. Like CathL, Cathepsin B is a lysosomal cysteine protease shown to have high expression in cancerous cells. The efficacy of the PNP to be preferentially activated in the tumor nucleus versus the cytoplasm of normal cells was demonstrated in vitro. Further, in HEYA8 xenograft and patient-derived xenograft mouse models, PNP showed greatly improved anti-tumor efficacy and significantly reduced adverse effects as compared to free DOX [124]. Similarly, arsenic trioxide lipid nanobins conjugated to uPA antibodies in murine OvCa models inhibited tumor growth by increasing cytotoxicity in the tumor cells versus untargeted nanobins, stressing the utility of targeting toxic agents to the protease microenvironment in OvCa [123].

Several works in the literature use protease sensitive polymeric nanocarriers. For example, nuclear localization targeting peptide linked with MMP-9 cleavable peptide and the antioxidant curcumin co-assembled with p53 DNA using cross-linked cationic polymer, polyethyleneimine (CUR-PEI-K14/p53), decreased CP resistance of SKOV3 cells in vitro [248]. The half-inhibitory concentration dropped from 10 ug/mL in cells treated with CP alone to 1 ug/mL in cells treated with CP+CUR-PEI-K14/p53. Further, while CP treatment did not induce p53 expression in the resistant cell model, the CP+CUR-PEI-K14/p53 treated cells did have increased p53 mRNA expression, suggesting the efficacy of the NP in overcoming resistance to CP. Another approach used was to modify the surface of NP based on proteolytic environment. PEGylation is used to create “stealth” NP with increased circulation time, based on the steric hindrance preventing their cellular uptake [249,250]. One study investigated Poly(l-glutamic acid)-CP (PLG-Pt) NP with detachable PEG conjugated to the surface with MMP-2/9-cleavable substrate PLGLAG (PEG-MMP-PLG-Pt). DePEGylation of these NP was triggered in the OvCa microenvironment, increasing tumor cell uptake of CP, while maintaining the PEG layer in normal tissues [251]. While the body weight of the free CP group fell, the weights were nearly unchanged in the PEG-MMP-PLG-Pt cohort, demonstrating the reduction in off-target side effects in addition to increased anti-tumor apoptotic activity and survival time.

**Table 3:** Examples of Protease Activated NP in OvCa.

Target Protease	Nanomedicine	Model	Outcome	Diagnostic
MMP-9	Iron Oxide NP core with tethered (a) PEGylated tumor penetrating ligand (LyP-1, CGNKRTRGC) and (b) PEGylated MMP substrate (PLGVRGK) with urinary reporter (NIR glutamate fibrinopeptide B)	OVCAR-8 orthotopic xenografts in nude mice	Detection of sub centimeter OvCa by MMP-9 cleaved urinary reporter; ROC-AUC(week 2) = 0.99 vs. HE4 biomarker ROC-AUC (week 2) = 0.51	[121]
Lentiviral induced upregulated Tobacco Etch Virus (TEV) Protease	40 kDa eight-arm PEG NP cojugated to TEV substrate (Biotin-eGvndneeGffsar-K(FAM)-dGGENLYFQGGGC) with urine reporter molecule (NIR glutamate fibrinopeptide B) or blood fluorescent marker	OVCAR8 IP xenografts in nude mice	Synthetic gene circuit coupled with NP readout to detect ovarian cancer via TEV Protease cleaved blood and urine reporter	[240]
				**Theranostic**
MMP-9	Liposomal core, PLR, luciferase siRNA, and pPLD layer-by-layer NP with azide-functionalized MMP-9 biosensor peptide (sequence B(biotin)-eGvndneeGffsarK-(FAM) dGGPLGVRGKK-(N3)), mPEG-azide, and Azide functionalized iRGD	OVCAR-8 orthotopic xenografts in nude mice	54% luciferase knockdown, detection of OvCa xenografts at an average 36 mm^3^ volume	[122]
				**Therapeutic**
MMP-9	Polymer drug conjugate of polyethyleneimine (PEI) cross linked with bifunctional tumor targeting and nuclear localization signal peptide (K14) and coupled to curcumin (CUR) with MMP-9 cleavable peptide (CPLGIAG) co-assembly with p53 (CUR-PEI-K14/p53)	SKOV3 carboplatin resistant cells (in vitro)	Increased transfection of p53 in CUR-PEI-K14/p53 versus PEI/p53 alone. Dose-dependent cytotoxicity of CUR-PEI-K14/p53 and decreased CP resistance with the addition of the polymer conjugate.	[248]
MMP-2/9	Nanocomplexes of PLG-CP with detachable PEG conjugated to pH (pHe)-responsive 2-propionic-3-methylmaleic anhydride-derived amide bond OR MMP-cleavable peptide PLGLAG (PEG-pHe-PLG-Pt and PEG-MMP-PLG-Pt)	BALB/c nude mice with IP OVCAR8 xenograft	Overcome steric repulsion of PEG at the site of tumor to increase intratumoral uptake of CP and improve anti-tumor activity	[251]
Cathepsin B	Self-assembling drug conjugate of Cathepsin B-specific cleavable peptide (FRRG) and DOX stabilized with pluronic F68 (termed PNPs)	BALB/c nude mice with HEYA8 IP xenografts (POX) OR BALB/c with platinum-resistant patient-derived subrenal capsule xenografts (PDX)	PNP increased IC_50_ in normal tissue cell culture of PNP vs.free DOX, suggesting minimizing off-target effects; PNP showed decreased major organ absorption and increased persistence in the peritoneal cavity vs. free DOX; PNP showed enhanced tumor penetration vs. free DOX; PNP treated mice had prolonged survival over 30 days vs. free DOX which had death at 19 days due to chemotoxicity	[124]

#### 4.4.2. Protease-Activity Driven Nanoparticle Interventions in Other Cancer Types

There are numerous examples of protease-powered nanomedicine interventions for other cancer types. For instance, azademethylcolchicine, a vascular disrupting agent that is utilized against solid tumors but has the untoward side effects of cardiotoxicity, has been developed into a MMP-14 activatable form termed ICT2588 (ICT) that avoids the unfavorable side effect profile by selectively activating in tumor tissues [252]. In a murine glioblastoma model, the synergistic activity of novel MMP-14-activatable cross-linked SPION conjugated to ICT (CLIO-ICT) with temozolomide caused significant apoptosis of cancer cells. These findings correlated with significantly prolonged survival of pcGBM39-bearing mice and complete tumor remission of pcGBM2-bearing mice. Further, the iron core had the dual function of improving MRI T2 darkening or negative enhancement in CLIO and CLIO-ICT-treated animals, respectively [247]. SPION with protease-affinity may be highly useful in OvCa diagnosis, treatment, and evaluation of treatment response.

Similar MMP-14 targeting has been demonstrated to improve cytoreductive surgery in childhood neuroblastoma by the creation of a FRET nanoprobe conjugated to MMP-14 peptide, in which cleavage of the peptide results in tumor illumination. Utilizing this technology allowed for an increase in positive tumor excision rate versus naked eye surgery in murine model (84% vs. 62%). This nanoprobe utilized Near Infrared (NIR)-II-emitting Ag_2_S quantum dots with (a) PEGylated AF7P, a peptide targeting MMP-14 loop domain in neuroblastoma to allow for primary localization and (b) NIR-II absorbing A1094 poly-anionic fragments (E8) conjugated to MMP-14 activated peptide, which conceals R9 cell penetrating peptide (TAT) prior to activation [253]. Once the nanoprobe reaches the TME, MMP-14 cleaves the substrate to release the E8 NIR-II absorber and TAT is revealed, causing tumoral uptake of the visibly fluorescent Ag_2_S quantum dots for improved cytoreduction. Translating such a technique to surgical debulking of OvCa is highly desirable, as identification of tumor margins and metastatic loci during the surgery is frequently challenging.

MMP remain a major focus of much protease-activated nanomedicine research. However, other previously discussed proteases have also been identified as targets for protease-sensitive intervention. In breast cancer models, poly(lactic-co-glycolic acid) (PLGA) and poly(lactic acid) (PLA) particles loaded with DOX with near-infrared fluorophores conjugated to a trypsin-cleavable polypeptide poly-l-lysine (PLL) linker, improved cancer-killing while simultaneously allowing for protease-activated imaging [254]. Further, macrophages in breast cancer upregulate AEP, a property which He et al. leveraged to create monocyte-delivered AEP-activated NP to deliver a chemotherapeutic payload to breast cancer lung metastasis [255]. Bossmann et al. demonstrated the ability to stabilize liposomes against osmotic pressure by the addition of a uPA sensitive cross-linked polymer shell, which burst open on exposure to uPa, a technology which could decrease of-target side effects of chemotherapeutics for multiple tumor types [256]. Thus, ample opportunity exists to investigate non-MMP protease-sensitive NP that may prove beneficial in OvCa diagnosis and treatment.

## 5. Conclusions and Future Directions

OvCa represents a significant clinical challenge and there has been a limited improvement in 5-year survival rates during the past decades. Due to its asymptomatic nature and inability of the currently available diagnostic/screening methods to detect the disease in early stages, the vast majority of cases are diagnosed when OvCa has disseminated. No effective techniques for early stage OvCa detection or reliable prognostic biomarkers for predicting therapeutic responses and shaping drug regimens are available today. Thus, novel tools for OvCa detection and management are highly required. The protease microenvironment in malignancy is vastly different from a healthy tissue and can be leveraged as a targeting mechanism for nanomedicines in OvCa. Being on the interface of cellular and molecular scales, NP can efficiently exploit biological cues in the tumor microenvironment, while protecting the cargo en route to the target tissue and minimizing adverse effects in healthy tissues. To this end, tumor penetrating nanosensors leveraging protease sensitive substrates have been shown to detect clinically undetectable tumors, and protease targeted nanotherapeutics enabled a more efficient and less toxic OvCa management. While molecular characteristics of protease-activated NP open new arenas for exploring early diagnostic and therapeutic tools in OvCa, it is important to remember that imparting various degrees of complexity to nanomedicine (e.g., NP clusters, targeting ligands, enzyme substrates, etc.) may hinder scaling up the fabrication and regulatory processes. Further, while nanomedicines have the potential to limit off-target side effects, improved efficacy against disease is not always realized in clinical metrics. On the other hand, the advantages that these agents may offer in OvCa warrant pursuing them to benefit OvCa patients in the future. Additional considerations in designing protease enabled nanomedicine include enhancing specificity and understanding and fine-tuning biodistribution and toxicity profiles. Continued innovation and translation of the protease targeting nanomedicines from the bench to the bedside is of great interest.

## Figures and Tables

**Table 1:** Staging of OvCa, adapted from the International Federation of Gynecology and Obstetrics (FIGO) and 5 year survival rate across all OvCa subtypes [5,7,8,9].

Stage	TNM Classification	5-Year Survival Rate
Stage I: Tumor does not extend beyond ovary/ovaries or fallopian tube/s	T1-N0-M0	Localized: 93%
IA: Tumor limited to single ovary or fallopian tube, the tumor capsule intact, and peritoneal washings free from malignancy	T1a-N0-M0	
IB: Tumor limited to bilateral ovaries or fallopian tubes, the tumor capsule intact, and peritoneal washings free from malignancy	T1b-N0-M0	
IC1: Tumor capsule is ruptured intraoperatively	T1c1-N0-M0	
IC2: Tumor capsule ruptured before surgery or tumor extends beyond the capsule to the ovarian/fallopian tube surface	T1c2-N0-M0	Regional: 74%
IC3: Presence of cancerous cells in ascites/peritoneal washings	T1c3-N0-M0	
Stage II: Unilateral/Bilateral Ovary/Fallopian tumor extends below the pelvic brim, or peritoneal cancer	T2-N0-M0	
IIA: Involvement of uterus and/or ovaries and/or fallopian tubes	T2a-N0-M0	
IIB: Other extension of tumor below pelvic brim	T2b-N0-M0	
Stage III: Unilateral/Bilateral Ovary/Fallopian tumor, or peritoneal cancer, that extends above the pelvic brim, and/or has confirmed metastasis to the retroperitoneal lymph nodes (RPLN)	T1-3/N0-1/M0	
IIIA1: Positive for metastasis to the RPLN only (proven by cytology/histology) IIIA1 (i) Metastasis ≤ 10 mm IIIA1 (ii) Metastasis ≥ 10 mm	T1/T2-N1-M0	
IIIA2: Microscopic peritoneal metastasis above the pelvic brim +/− positive RPLN	T3a2-N0/N1-M0	Distant: 30%
IIIB: Macroscopic peritoneal metastasis beyond the pelvis ≤ 2 cm +/− metastasis to the RPLN	T3b-N0/N1-M0	
IIIC: Macroscopic peritoneal metastasis beyond the pelvis ≥ 2 cm, including non-parenchymal extension to liver and/or spleen, +/− metastasis to the RPLN	T3c-N0/N1-M0	
Stage IV: Distant metastasis	Any T, any N, M1	
Stage IVA: Pleural effusion with cytology positive for malignant cells	T_-N_-M1	
Stage IVB: Parenchymal metastases to liver and/or spleen and metastases to extra-abdominal organs, inguinal lymph nodes and/or lymph nodes outside of the abdomen		

**Table 2:** Summary of Selected Proteases in OvCa Tumorigenesis and Metastasis.

Protease	Family	Summary of Role in Tumorigenesis and Metastasis	Select Sources
MMP-2	Soluble Metalloproteinase	Angioproliferative by increased VEGF	[52]
MMP-9	Soluble Metalloproteinase	ECM remodeling by degradation of E-cadherin, basement membrane; Angioproliferative by increased VEGF	[53,54]
MMP-14	Membrane Bound Metalloproteinase	Angioproliferative by increased VEGF; remodeling of collagen	[55,56]
uPA	Serine Protease	Activates plasmin to promote ECM remodeling; increases neovascularization; prevention of tumor cell apoptosis	[57,58,59]
Trypsin	Serine Protease	Activates uPA; ECM remodeling; Activates MMP-9; Increases cellular proliferation	[60,61,62,63,64]
PAPP-A	Zinc Metalloproteinase	Cell proliferation via IGF upregulation	[65,66,67,68,69]
CathL	Cysteine Protease	Angiogenesis, inflammation, ECM remodeling; invasion, metastasis, increased cellular proliferation/inhibition of cellular apoptosis	[70,71]
CathD	Aspartic Protease	Activates plasmin and IGF; Increased activity of uPA; inhibition of Cystatin C (CathL inhibitor) to allow for increased CathL activity; ECM degradation, angioproliferative, natural killer cell evasion via mesenchymal stem cell chemoattraction	[72,73,74,75,76]
KLK4	Serine Protease	Activates uPA, KLK-5, KLK-6; ECM remodeling and metastasis by increased TGF-B1 and L1 CAM expression	[77,78]
AEP	Cysteine Protease	Pro-peritoneal metastasis via activation of the FAK/AKT/ERK signaling pathway; activation of MMP-2 and MMP-9	[79,80,81,82]
